# Home Care Providers to the Rescue: A Novel First-Responder Programme

**DOI:** 10.1371/journal.pone.0141352

**Published:** 2015-10-28

**Authors:** Steen M. Hansen, Stig Brøndum, Grethe Thomas, Susanne R. Rasmussen, Birgitte Kvist, Anette Christensen, Charlotte Lyng, Jan Lindberg, Torsten L. B. Lauritsen, Freddy K. Lippert, Christian Torp-Pedersen, Poul A. Hansen

**Affiliations:** 1 Department of Health, Science and Technology, Aalborg University, Aalborg, Denmark; 2 Hjerteforeningen, Danish Heart Foundation, Copenhagen, Denmark; 3 The Danish Foundation TrygFonden, Copenhagen, Denmark; 4 KORA, Danish Institute for Local and Regional Government Research, Aarhus, Denmark; 5 Department of Health and Nursing, Municipality of Frederikshavn, North Denmark Region, Frederikshavn, Denmark; 6 Prehospital Care Organization, North Denmark Region, Aalborg, Denmark; 7 Home Care Organization, Municipality of Frederikshavn, North Denmark Region, Frederikshavn, Denmark; 8 Department of Anaesthesia, The Juliane Marie Centre, Rigshospitalet, University Hospital of Copenhagen, Copenhagen, Denmark; 9 The Emergency Medical Services, Copenhagen, Denmark; Azienda Ospedaliero-Universitaria Careggi, ITALY

## Abstract

**Aim:**

To describe the implementation of a novel first-responder programme in which home care providers equipped with automated external defibrillators (AEDs) were dispatched in parallel with existing emergency medical services in the event of a suspected out-of-hospital cardiac arrest (OHCA).

**Methods:**

We evaluated a one-year prospective study that trained home care providers in performing cardiopulmonary resuscitation (CPR) and using an AED in cases of suspected OHCA. Data were collected from cardiac arrest case files, case files from each provider dispatch and a survey among dispatched providers. The study was conducted in a rural district in Denmark.

**Results:**

Home care providers were dispatched to 28 of the 60 OHCAs that occurred in the study period. In ten cases the providers arrived before the ambulance service and subsequently performed CPR. AED analysis was executed in three cases and shock was delivered in one case. For 26 of the 28 cases, the cardiac arrest occurred in a private home. Ninety-five per cent of the providers who had been dispatched to a cardiac arrest reported feeling prepared for managing the initial resuscitation, including use of AED.

**Conclusion:**

Home care providers are suited to act as first-responders in predominantly rural and residential districts. Future follow-up will allow further evaluation of home care provider arrivals and patient survival.

## Introduction

The prompt delivery of cardio-pulmonary resuscitation (CPR) and early defibrillation before the arrival of emergency medical services (EMS) in the event of an out-of-hospital cardiac arrest (OHCA) are considered among the most important determinants of survival [1–3]. Bystanders can safely operate automated external defibrillators (AEDs) placed in public locations with high incidence of OHCAs, thus reducing time to first defibrillation and increasing survival [2,4–7]. As a result, recent years have seen a worldwide dissemination of AEDs [4,5,8–10]. However, as most OHCAs occur in private homes and not only in high density population areas, early defibrillation is hampered by availability and effective coverage of stationary AEDs [3,11,12]. Patients in private or rural areas thus continue to depend primarily on EMS and first-response programmes for early defibrillation.

Various first-responder programmes involving for example fire fighters, police officers and/or local lay rescuers have been described [13–19]. Although such programmes have typically been conducted in urban and suburban areas [20], reports from first-responder programmes in rural districts have been encouraging, especially with respect to reducing time-to-arrival at the cardiac arrest site [21,22].

In Denmark, the care of frail and dependent elderly people is widely performed by home care providers travelling by car to visit people in their own homes. This article describes a one-year experimental programme for the provision of first response in OHCA cases by home care providers working in a Danish municipality with mixed urban and rural settlement. After systematic training in performing basic life support including the use of an AED, the providers were dispatched in parallel with conventional EMS to cardiac arrest incidences.

## Methods

### Setting

The study was conducted in the municipality of Frederikshavn in north Denmark between October 1, 2012 and September 30, 2013 [23]. Covering 649 square kilometres, Frederikshavn had 61,119 inhabitants by early 2012. The municipality is mainly rural, with scattered villages, farmed land and three urban centres, Frederikshavn, Sæby and Skagen, with approximately 23 000, 9 000 and 8 500 inhabitants, respectively [24].

When the emergency medical dispatch (EMD) centre covering the municipality is alerted about a suspected OHCA incidence, a two-tier dispatch system is activated. The system comprises basic life support provided by emergency medical technicians in defibrillator-equipped ambulances and advanced life support delivered by paramedic staff in an emergency vehivle. When available, a physician-staffed rapid response car was also dispatched.

All resuscitated OHCA patients are referred to the nearest emergency hospital (Aalborg University Hospital), a distance of 50–100 km, for advanced post-resuscitation care.

Average ambulance response times for Frederikshavn were 9:29 and 9:04 (min:sec) in 2012 and 2013, respectively.

It was expected that home care providers would be dispatched to about 50% of 65–70 suspected OHCAs during the study period.

### Home care provision

In Denmark, home care providers are employed by the municipality to visit residents in need of care to support their continued living in their own homes. The providers’ background education ranges from basic seven-year to upper secondary schooling. The majority have received vocational training in basic care. Different tasks are performed by the providers depending on their professional training, from assistance with cleaning and personal hygiene to medicine dispensation by registered nurses.

Before initiation of the dispatch programme, 614 providers followed a 2.5-hour training course in performing CPR and using an AED. The course was organized by the Emergency Medical Services of North Denmark Region, one of Denmark’s five regional authorities tasked with the provision of health care. The course was based on European Resuscitation Council recommendations, each sequence involving 7–20 participants [25]. In addition, the providers received 30 min of instructions in handling the smartphone dispatch system. Weekly test calls and monthly dispatches maintained acquired skills [26].

### Equipment and technology

All of the approximately 60 home care provider cars were equipped with a Zoll AED Plus system including a kit containing pads, scissors, gloves and cardiac-arrest action cards. The cars were also equipped with a Samsung GSM Galaxy S Plus smartphone with a bespoke “SimaGo” application (SimaTech, Brondby, Denmark) that allowed the EMD to GPS-track and alert the providers by a text message. Upon SimaGo activation the two providers nearest to the OHCA site were informed of the patient’s location, with the option of accepting or declining the call. The providers were instructed to decline a call if they were unable to interrupt their ongoing task, in which case the third-nearest provider was activated, and so forth. If no response was received from an activated provider within 35 s, the call was automatically registered as declined.

### Dispatch conditions

When an OHCA was suspected, home care providers were alerted by the EMD and dispatched in parallel with conventional ambulance services according to the study protocol. In the following cases, the study protocol mandated no alert of home care providers: i) the suspected OHCA patient was younger than seven years of age, ii) the cause of the suspected OHCA was due to trauma/suicide or poisoning, iii) when obvious signs of death were reported to the EMD, iv) the OHCA was witnessed by ambulance services or v) the home care provider was further than 10 km away from the OHCA site. The home care providers were instructed to strictly observe speed limits and other traffic regulations, when alerted. The first provider to arrive was instructed to focus on performing CPR; on the arrival of a second provider, an AED was to be applied.

### Data collection

The ambulance personnel dispatched to a suspected OHCA were required to complete a case file describing the event. The home care providers also completed a case file for each dispatch, including times of SimaGo activations, arrival times and descriptions of the tasks performed during the OHCA incident. We manually reviewed all files reported for this study to confirm actual cardiac arrest, validate whether the provider had arrived before ambulance services, and obtain details including response time and patient status at hospital arrival. Initially, the providers’ arrival times proved unreliable, either because of inaccurate manual registrations by the provider or malfunctioning GPS tracking. The GPS-related problems were corrected in a system update by mid-February 2013. Consequently, response times and distances to the provider nearest to the OHCA site were only available from that time. In addition to the case file, the providers were asked to complete a questionnaire after being dispatched. The questionnaire elicited information on the cardiac arrest, the tasks performed by the provider, any cooperation with the ambulance services, and self-evaluation of preparedness in handling the OHCA situation. The latter was covered by responses to a four-question scale with the options “largely, “to some extent”, “to a lesser extent”, and “to a small extent”.

### Data Analysis

We used Pearson’s chi-squared test to evaluate differences in categorical variables and the Kruskal-Wallis rank sum test to evaluate differences in non-normally distributed continuous variables. A two-sided P-value<0.05 was considered statistically significant.

Data management and analysis were performed using SAS 9.4 and R version 3.1.1 software (SAS Institute Inc. and R Development Core Team, respectively).

### Ethics

The Danish Data Protection Agency (J.nr.: 2013-41-1844) approved this register-based study, which required neither ethical approval by the local ethics committee nor written informed consent.

## Results

**Fig 1:**
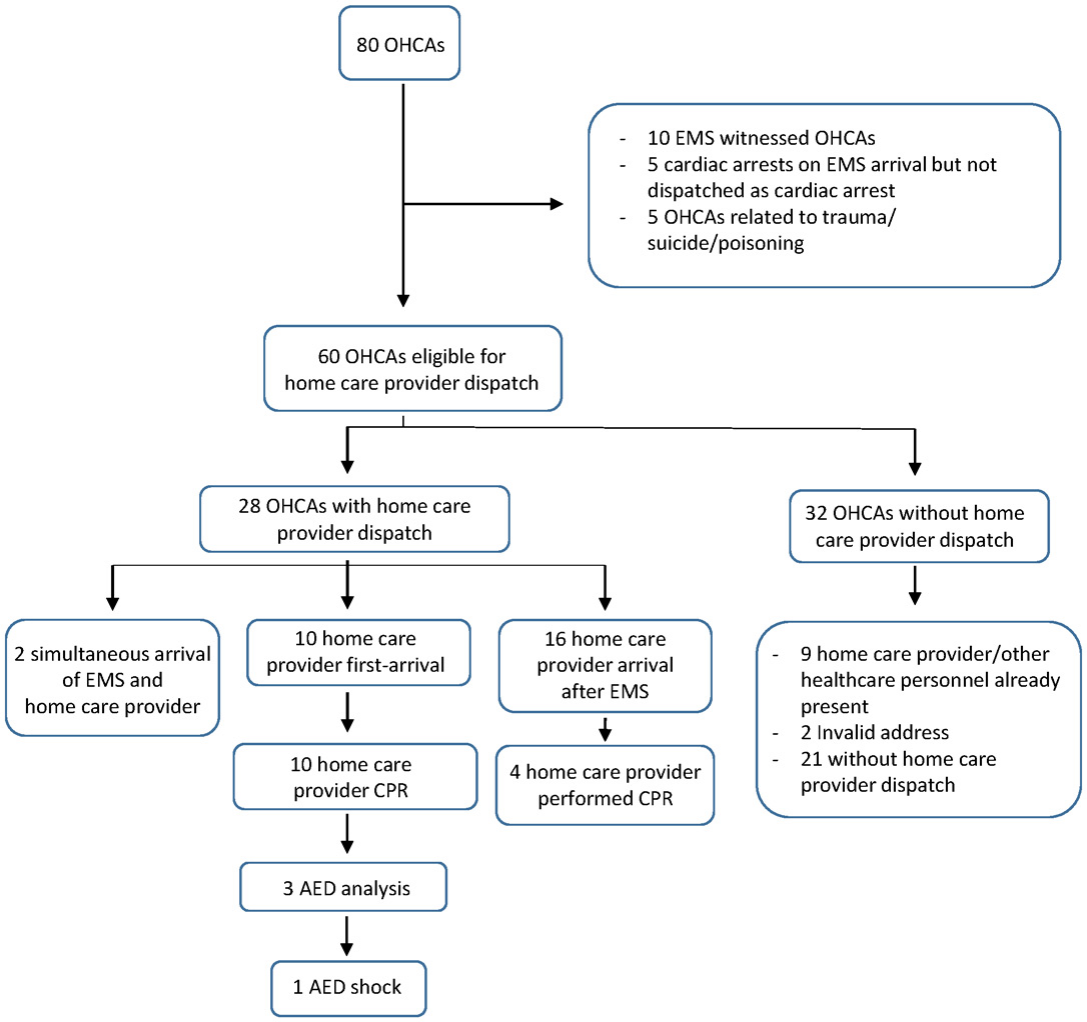
OHCA study population in home care provider first-responder programme. OHCA = Out-of-hospital cardiac arrest. EMS = Emergency medical services. CPR = Cardiopulmonary resuscitation. AED = Automated external defibrillator.

Eighty OHCAs occurred during the one-year study period. While the dispatch of home care providers was a relevant option in 60 cases (Fig 1), the providers were dispatched in 28 cases (47%). For ten of those dispatches (36%), a home care provider was the first to arrive and perform CPR. AED analysis was performed in three of the ten instances (30%), and one shock was delivered. In three additional cases of the first-arrivals, a home care provider had begun preparing defibrillation (AED), but analysis was not achieved before EMS arrived. In 16 of the 28 dispatches (57%), a home care provider arrived after EMS while simultaneous arrivals occurred in two cases (Fig 1). In the remaining 32 OHCA cases, no home care provider was dispatched. In nine cases, the reason was that other healthcare personnel was already present at the site. In two cases, the address was unknown. Various other reasons were cited for the remaining 21 cases.

Table 1 shows characteristics for all registered OHCA cases, with or without home care provider being dispatched, with various subgroups. No differences in age (P = 0.21), sex (P = 0.43) or patient status at hospital arrival between groups were detected (P = 0.71). When a home care provider arrived before ambulance services, the median ambulance response time was 9 min compared to 5 min and 6 min when a home care provider arrived after or simultaneously with ambulance services, respectively. The median ambulance response time was 4 min in cases where no home care providers were dispatched.

**Table 1:** Out-of-hospital cardiac arrest characteristics.

	Dispatched			Not dispatched					
	Home care provider first arrival	EMS first arrival	Simultaneous arrival	No home care provider dispatch	Healthcare personnel present at site	Invalid address	Total	Missing data	P-value
	n = 10	n = 16	n = 2	n = 21	n = 9	n = 2	n = 60		
**Age**									
Median age in years {IQR}	72 {72, 80}	71 {64, 76}	70 {68, 73}	71 {53, 77}	84 {78, 90}	72 {69, 76}	74 {65, 80}	2	0.21
**Sex**									
Men	6 (60)	12 (80.	1 (50)	15 (71)	4 (44)	2 (100)	40 (68)		
Women	4 (40)	3 (20)	1 (50)	6 (29)	5 (56)	0 (0)	19 (32)	1	0.43
**Patient status at hospital arrival**									
Dead	7 (70)	9 (60)	1 (50)	13 (62)	7 (78)	2 (100)	39 (66)		
Continued resuscitation attempt	1 (10)	1 (7)	1 (50)	3 (14)	0 (0)	0 (0)	6 (10)		
Achieved ROSC	2 (20)	5 (33)	0 (0)	5 (24)	2 (22)	0 (0)	14 (24)	1	0.71
**EMS response time**									
Median time (min:sec) {IQR)	9:00 {6:00, 12:00}	5:00 {4:00, 9:00}	6:00 {4:00, 8:00}	4:00 {4:00, 9:00}	9:00 {4:00, 13:00}	13:00 {13:00, 13:00}	05:00 {4:00, 11:00)		0.11
**Home care provider response time** *									
Median time (min:sec) {IQR}	5:37 {5:25, 7:28}	11:03 {5:40, 12:26}	6:37 {5:02, 8:12}	-	-	-	7:41 {5:22, 11:27}	1	0.23
**Nearest home care provider distance to OHCA site** *									
Median distance (meters) {IQR}	640 {591, 840}	988 {626, 2982}	1666,5 {389, 2944}	-	-	-	840 {389, 2785}		0.65

All results are n (%) unless otherwise specified. IQR = Interquartile range EMS = Emergency medical service OHCA = Out-of-hospital cardiac arrest

* Only OHCA cases from mid-February 2013 until September 30 2013 (n = 21)

During the study period, 46 home care provider dispatch calls were executed from the EMD. Two calls were unintentional as the OHCA occurred outside the study municipality. Four calls failed, one caused by call system malfunction, one because no home care provider was within ten km of the OHCA site and two calls were rejected by the providers. In 91% (40/44) of cases, the call was accepted by at least one home care provider who subsequently arrived at the site. For 12 of the dispatches, no resuscitation attempts were performed as six patients did not have cardiac arrest and six were found with obvious signs of death.

Table 2 summarizes and compares the 40 dispatch calls. The age of patients without cardiac arrest were lower than patients with cardiac arrest (P<0.001). Both in dispatches to cardiac arrests and to other cases, the majority of patients were men. In 19 of the 28 dispatch cases (68%) two home care providers arrived.

**Table 2:** Home care provider dispatch calls.

	Cardiac arrest	No cardiac arrest	Total	Missing data	P-value
	n = 28	n = 12	n = 40		
**Age**					
Median age {IQR}	74 {68, 78}	54 {48, 64}	69 {59, 76}	1	<0.001
**Sex**					
Men	19 (70)	9 (75)	28 (72)		
Women	8 (30)	3 (25)	11 (28)	1	1
**Number of HCPs who arrived**					
One	9 (32)	6 (50)	15 (38)		
Two	19 (68)	6 (50)	25 (62)		0.48
**Location**					
Private home	26 (93)	12 (100)	38 (95)		
Public location	2 (7)	0 (0)	2 (5)		0.87
**Dispatch Delay**					
Median time (min:sec) {IQR}	2:23 {1:52, 3:01}	2:21 {1:44, 3:38}	2:23 {1:46, 3:38}	1	0.99
**Preparedness for handling the OHCA situation** *					
Largely/to some extent	39 (95)	-	39 (95)		
Lesser/to small extent	2 (5)	-	2 (5)	6	-

All results are n (%) unless otherwise specified. IQR = Interquartile range. HCP = home care provider.

OHCA = Out-of-hospital cardiac arrest.

* 41 post-dispatch questionnaires were adequately answered by providers.

The average delay before home care providers were activated was 2 min 20 s, as counted from the receipt of the emergency call by EMD (no difference between groups; P = 0.99).

### Residential locations

In 26 of the 28 OHCA cases (93%), the cardiac arrest occurred in a private home. In all OHCA cases where a home care provider arrived before EMS, the cardiac arrest happened in a private home. In 95% of all dispatches, the home care providers were dispatched to a private home (Table 2).

### Home care providers’ self-evaluation of preparedness

Following dispatches, the home care providers were asked to indicate their preparedness for handling the situation. Forty-two questionnaires were answered by 47 providers who had met with cardiac arrest caseS (89.4%). No questionnaire forms were obtained for four arrest cases involving six providers. All but one of the 42 forms were sufficiently completed. In total, 39 of 41 providers (95%) reported being “largely” or “to some extent” prepared for being dispatched to a cardiac arrest patient and managing initial resuscitation (Table 2).

## Discussion

This article describes the implementation of a novel AED first-responder programme concerning the dispatch of home care providers in parallel with conventional EMS in cases of suspected OHCA. The activation of the providers was EMD-controlled by the use of smartphones with bespoke equipment.

Previous studies have described programmes in which laypersons, police officers and fire-fighters have acted as first-responders in the event of a nearby OHCA to provide CPR and early defibrillation[14,16,21,22,27–30], but the presence of such groups can be low in sparsely populated rural districts. In contrast, mobile home care providers are working locally around the clock to support elderly and other citizens in need of care, and their deployment as first-responders to OHCAs could prove valuable. In this study, the providers were given systematic training in the use of AED and to perform CPR in a 2.5-hour course followed by 30 min of instruction in utilizing the smartphone dispatch system.

With the successful dispatch of home care providers in almost half of all suspected OHCA cases and their arrival before ambulance services in more than a third of the cases, the expected rate as assessed before the study was reached. In addition, our results are comparable to those of previous first-responder studies. In a study considering laypersons as first-responders the responders were dispatched to 58% of OHCA cases [22]. A study involving fire-fighters as first-responders reported that the first-responders were dispatched in 66% of all OHCA cases and arrived before EMS in 36% of all dispatches [14]. A study of police officers reported dispatch to 54% of cardiac arrests and arrival before EMS in 16% of cases [16]. Another study recruiting both laypersons and police officers as first-responders in rural areas reported dispatch to 40% of all OHCA events. Moreover, the mixed group arrived before EMS in 47% of the dispatches [21].

In three of the cases (30%) in which the home care providers in our study were the first to arrive, they performed AED analysis and in one case AED shock was delivered (10%). In three other cases, a home care provider had begun preparing an AED but did not complete AED connection before ambulance arrival.

A previous first-responder study has reported that an AED was connected in 43.5% of OHCA cases in a rural area [21]. Another study reported that the AED had been connected before EMS arrival for 23.1% of dispatches [22]. In our study, the first home care provider to arrive had instructions to focus on performing CPR and apply AED only after the arrival of a second provider; CPR was thus performed in all first arrivals. In two cases, a provider would have been activated if the SimaGo system had allowed providers to be dispatched to non-address locations, an option that should be considered for future first-responder systems.

A short response time of ambulances was the typical reason for non-dispatch of home care providers. It has been recommended that first-responders should be dispatched irrespective of EMS response times [22]. We cannot rule out that in spite of the low ambulance response time, the providers would have arrived before the ambulance if they had been alerted. However, when they did arrive before the ambulance, the median ambulance response time was 9 min as opposed to 5 min when an ambulance arrived first. Thus, the chance of provider first-arrival seems limited when an ambulance is nearby.

The cars used by the providers in our study had no siren. Because of the acknowledged risk of traffic accidents, it was stressed that all traffic regulations were to be followed strictly. No traffic accidents or safety issues were reported.

The delay of 2 min 20 s between the receipt of the emergency call by EMD and the dispatch of home care providers decreased the chance of first arrival. A previous study has reported delays of about 2 min, primarily caused by latency in identifying the emergency as a cardiac arrest [14]. First-responder programmes should give priority to minimizing such delays, for instance by alerting the providers when specific keywords are recognized by EMD personnel. However, such activation could increase the risk of providers being dispatched to non-cardiac arrest emergencies. In a study of first-responders who were dispatched not only to suspected OHCA cases but also to emergencies concerning unconscious patients, only 4.9% of all dispatches met with actual cardiac arrest cases as opposed to 70% in our study. However, despite their location in an urban area with short EMS response times, the first-responders in that study arrived before EMS in 73% of all cases [19].

Almost all OHCAs for which the home care providers in our study were dispatched occurred in a private home, as found in other studies as well [22]. We consider this a strength as most cardiac arrests occur in areas where AED availability is limited [3]. In the post-dispatch responses given by home care providers, 95% reported that they had felt largely or to some extent prepared to manage initial resuscitation. This is a very encouraging result that compares with or surpasses satisfaction results from other first-responder programmes that have studied fire-fighters, police officers and lay volunteers [31–33].

### Limitations

While our study was adequately powered to demonstrate feasibility and examine reactions from a new group of first-responders, it could not provide satisfactory results for efficacy. Consequently, we were unable to demonstrate any difference in status at hospital arrival or potential improvements in response time or time to defibrillation. However, the continuation of the home care provider first-responder programme allows us to perform follow-up.

The response rate for questionnaires after OHCA dispatches was 89.4%. A bias may have been introduced by the fact that single cases elicited more than one response regarding readiness. Furthermore, a selection bias cannot be ruled out as the respondents could identify themselves on the questionnaire forms. However, even if all non-responders were assumed to have felt unprepared in handling the cardiac arrest situation, the “preparedness” score would have been approximately 83%.

The response times reported for the beginning of the study period proved to be inaccurate. Response times and distances travelled by the home care providers do therefore not cover the whole study period. For the remaining time, we reviewed each case file to validate whether the home care providers had arrived before ambulance services.

## Conclusion

The studied home care providers were suited to act as first-responders. In residential and rural districts, home care providers could be considered for deployment as first-responders. Follow-up studies will allow further evaluation of home care provider arrivals and patient survival.
